# Math anxiety predicts initial acquisition of new math skills over and above math competence and study strategy

**DOI:** 10.3389/fpsyg.2026.1829468

**Published:** 2026-09-08

**Authors:** Amira F. A. Ibrahim, Priti Shah

**Affiliations:** 1 Department of Psychology, California State University, Channel Islands, Camarillo, CA, United States; 2 Department of Psychology, University of Michigan, Ann Arbor, MI, United States

**Keywords:** math achievement, math acquisition, math anxiety, math performance, practice problems, worked examples

## Abstract

**Introduction:**

Math anxiety is negatively associated with math performance and achievement. However, we do not know much about how math anxiety relates to the acquisition of new math skills. The current study aims to investigate (1) whether math anxiety is negatively associated with the acquisition of new math skills above and beyond baseline math competence and (2) if studying examples versus completing practice problems leads to different outcomes based on individual differences in math anxiety and basic math competence.

**Methods:**

Two hundred forty-nine college students were taught a novel mathematical computation and randomly assigned to four different study conditions consisting of varied numbers of worked-out examples and to be solved problems.

**Results:**

We found that individuals with higher math anxiety had lower performance on the novel math task than their less anxious counterparts, above and beyond their basic math competence.

**Discussion:**

Post-hoc mediation analysis suggests that math competence partially mediates the relationship between math anxiety and performance on the novel math task. The current study did not find compelling evidence to suggest differences between study strategies on acquisition of new math skills.

## Introduction

Math anxiety is commonly defined as “a feeling of tension and anxiety that interferes with the manipulation of numbers and the solving of mathematical problems in a wide variety of ordinary life and academic situations” (Richardson and Suinn, 1972, p. 551). Math anxiety is associated with lower math achievement, negative attitudes toward math, lower confidence in one’s math ability, and a decrease in the perception of the utility of math (Ashcraft and Kirk, 2001; Hembree, 1990; Li et al., 2021; Ma, 1999; Wigfield and Meece, 1988).

A preponderance of research on math anxiety addresses how math anxiety impairs math performance, which we define as the ability to implement existing knowledge or skills to solve math problems. On the one hand, deficits in basic numerical cognition and past low math performance are related to increases in math anxiety (Gunderson et al., 2018; Ma and Xu, 2004; Maloney et al., 2010; Maloney et al., 2011), suggesting that math anxiety may, in part, be a consequence of poor math competence (this is referred to as the reduced competency account of math anxiety). At the same time, math anxiety itself consumes cognitive resources via rumination or distraction, interfering with math performance (a disruption account; Ashcraft and Faust, 1994; Ashcraft and Moore, 2009; Faust, 1996). Indeed, it is likely that both math competence and cognitive depletion are relevant and that math performance and math anxiety have a reciprocal relationship (Ashcraft et al., 2007; Carey et al., 2016).

Much less is understood about how math anxiety interferes with the acquisition of new mathematics skills. Those with high math anxiety may have difficulty acquiring new math skills because they have lower math competence in the first place (e.g., experiencing difficulty with acquiring the relevant new knowledge due to lower pre-existing mathematical performance, achievement, and knowledge). In colloquial terms, they are building on a shakier foundation. However, math anxiety might also impact skill acquisition by consuming cognitive resources that are thus not allocated toward successfully navigating the phases of skill acquisition (which itself is resource demanding; Renkl, 2014). For example, if cognitive resources are limited, maintaining the procedural rules required to solve a problem becomes challenging. As with performance, both factors likely play a reciprocal role in acquiring new math skills.

The current study assesses whether math anxiety relates to the initial acquisition of a new mathematics skill above and beyond initial differences in math competence, and whether the study strategy used during acquisition interacts with math anxiety. Some prior research has examined the effect of math anxiety on the ability to acquire new math skills, such as verifying modular arithmetic problems (Bellinger et al., 2015; Legg and Locker, 2009) and completing a symbolic math task (Jenifer, 2021; Study 3). However, these studies do not control for math competence, mirror traditional skill acquisition common in the math classroom, and/or assess the role of varying study strategies. For example, the common modular arithmetic task used is a mental verification task, as in students are not solving problems but are assessing the accuracy of a provided problem in their mind. Generally in the classroom, students are provided with a lesson on the conceptual and abstract mathematical concept, then presented with examples of concrete applications of the concepts, and eventually attempt to apply this information to solve new problem sets themselves on paper (Renkl, 2014).

Insofar as we are aware, there are no peer-reviewed published studies exploring the relationship between math anxiety and new math skill acquisition that (1) account for individuals’ math competence, (2) use a task that is similar to what students would experience in the classroom, and (3) compares common skill acquisition strategies. Identifying the association between math anxiety and acquisition of novel math skills, above and beyond math competence, will clarify whether it merely interferes with the application of basic skills or if math anxiety potentially poses an additional burden on performing the novel math task. In addition, the manner in which the novel content is acquired, e.g., study strategies, could interact with math anxiety to either mitigate its negative effects or exacerbate them. Addressing these holes in the literature will allow for a better understanding of how to support those struggling with math anxiety. In the current study, we assess the extent to which math anxiety relates to new math skill acquisition, above and beyond individuals’ existing basic math competence, and explore whether the strategy used while studying the novel information interacts with the individuals’ math anxiety to achieve different outcomes.

### Math anxiety and acquisition of new math skills

Two published studies have explored the relationship between math anxiety and the acquisition of new mathematical skills in a more typical learning context. Tomasetto et al. (2021) explored the association between math anxiety and the learning of novel math content in elementary school children. They found that math anxiety was directly associated with lower performance on an immediate post-test of the novel content, and indirectly associated with a post-test at a one-week delay. Although they controlled for the children’s baseline performance on the novel task, they did not account for their preexisting math competence. Another study by Soltanlou et al. (2019) found that children with high math anxiety had impaired math learning, but only if they also had low visuospatial working memory. Children with high visuospatial working memory seemed to be able to compensate because they had adequate working memory capacity for the task, even if some resources were engaged in thinking about or controlling their math anxiety. This study also did not consider pre-existing mathematics ability, but it provides additional evidence that math anxiety may impair the learning of novel math skills.

To better understand the role of math anxiety in acquiring new math skills, it is important to consider the reciprocal relationship between math anxiety and performance, as discussed above. One way to do this is by accounting for individuals’ preexisting general math competence, not just their performance on a specific test or controlling for math achievement. Controlling for test scores addresses students’ competence on that particular task, but it does not reflect their broader math ability. This distinction is important for two reasons:

1. To determine whether math anxiety uniquely predicts performance on the novel task beyond what can be explained by general math competence.
2. To assess whether the observed relationship is specific to the new task or simply reflects the general disruptive effects of math anxiety on math performance.

In addition to controlling for math competence, it is also important to control for variables that are often correlated with math anxiety and/or math competence that could explain some variability in performance on a newly acquired math task, namely working memory capacity, test anxiety, and gender. Working memory capacity plays an important role in math performance because it is associated with the number of mathematical rules and procedures one is able to maintain online as they reason and solve problems (Ramirez et al., 2013; Soltanlou et al., 2019). In addition to controlling for working memory, other variables important to consider are gender and test anxiety. Test anxiety is highly correlated with both math anxiety and math performance (Foley et al., 2017; Ganley and Vasilyeva, 2014), and several studies have suggested that women report higher math anxiety than men (Dowker et al., 2016; Szczygieł et al., 2025). Controlling for these factors, in addition to general math competence, helps isolate unique variance accounted for by math anxiety on new math skill acquisition.

### Study strategies and math performance

In addition to general math competence, how novel math skills are acquired could also be an important component of the relationship between math anxiety and the acquisition of novel math skills. Learning new material is generally cognitively demanding, but some learning strategies are thought to burden cognitive resources less than others, making them more conducive to learning by facilitating the formation of schemas (Renkl, 2014; Sweller et al., 1998). This can be particularly important for individuals who already have constraints on their cognitive resources, such as those with math anxiety (Mesghina et al., 2024). Two common learning strategies are studying worked examples of problems and solving practice problems. Worked examples are example problems that are presented with a step-by-step breakdown that is used for study (Renkl, 2014). Theoretically, worked examples are thought to reduce cognitive load because one does not need to try to remember all the relevant information, and the individual needs only to study the example. The benefit of studying worked examples can be more pronounced under certain circumstances. For instance, some studies have found an interaction between worked examples and individual expertise (Cooper and Sweller, 1987; Kalyuga et al., 2001; Tuovinen and Sweller, 1999), such that worked examples were more beneficial for individuals with low previous knowledge. It is possible that the use of worked examples could also have differential effects on performance based on other individual differences, such as math anxiety. Mesghina et al. (2024) found that worked examples mediated the relationship between math anxiety and math learning, but they did not compare the use of worked examples to an alternate learning strategy such as problem solving. So although worked examples seem to have some benefit for those with math anxiety, it is still not clear whether they are beneficial above and beyond alternate learning strategies.

In contrast, some evidence suggests that more effortful learning strategies lead to better learning and long-term retention, deemed “desirable difficulties” (Bjork, 1994; Bjork and Bjork, 2020). The education and memory literature has long provided support for constructivist learning approaches, in which learning is thought to occur best when students are actively constructing their own knowledge instead of being passive receivers of information, despite it being more cognitively demanding (Bertsch et al., 2007; DeCaro and Rittle-Johnson, 2012; Rittle-Johnson and Kmicikewycz, 2008; Slamecka and Graf, 1978; Wheatley, 1991). One example is the generation effect, a phenomenon in which information that is generated is better remembered than information that is passively read (Slamecka and Graf, 1978). Bertsch et al. (2007) found that generation benefited recall by almost half of a standard deviation (0.40). They also found that the generation effect increased with increasing retention intervals and was more beneficial for difficult material, likely because generation helps curb the normal rate of forgetting that generally occurs when simply reading materials. The vast majority of studies have explored the generation effect in verbal recall; however, there have been several studies illustrating that the benefit of generation during study extends to math material as well (McNamara and Healy, 1995, 2000; Rittle-Johnson and Kmicikewycz, 2008). Rittle-Johnson and Kmicikewycz (2008) found that students with low previous knowledge (based on pre-test performance) in the generate condition benefited more than those who used a calculator during study. However, those with high previous knowledge performed similarly well in both conditions. This study suggests that actively practicing math material during study might be especially important for students with low previous knowledge. In summary, active construction of to-be-learned information has been shown to enhance performance for math material.

### The current study

The existing research illustrates some of the ways math anxiety interferes with performance but does not provide evidence as to the effects of math anxiety on the acquisition of new math skills. In addition, it is unclear whether study strategies may interact with math anxiety to influence acquisition. The current study seeks to fill some of these holes in our existing understanding of math anxiety and its relationship with the acquisition of new math skills and the effectiveness of different study strategies for individuals who vary in math anxiety. Notably, the current study uses a novel math acquisition task and controls for three key factors: math competency, test anxiety, and visuospatial working memory.

The current study explores the following research questions:

1. Does math anxiety account for unique variance in performance of newly acquired math skills above and beyond general math competence?
2. Do different study strategies interact with math anxiety to predict the performance of newly acquired math skills?

In the following study, participants were taught to perform base number conversions; the procedure to convert a number in base 10 into an alternate base is called the remainder method. After being introduced to the topic with a brief video lesson, participants studied the material with one of four study strategies, which varied in the level of independent problem solving. Participants then took a five-minute break before completing an immediate test. Participants also completed measures of math anxiety, math competence, test anxiety, and visuospatial working memory.

## Methods

### Participants

Two hundred fifty-two undergraduate students from a large midwestern predominantly white institution consented to participate either for credit or for $20, but three participants chose not to continue after completing the demographics and math anxiety measure. The final sample consisted 249 participants that was 44.2% male (*n =* 110), and was an average age of 19.94 years (*SD* = 4.49). 51.8% of the sample were in their first year of college, 22.5% in their second, 12% in their third, and the remaining were further along. See Table 1 for last math course completed, and Table 2 for majors.

**Table 1:** Distribution of last math course taken in sample.

Last Math Course	% of Sample
Calculus 1 or higher	66.7
Pre-calculus or lower	16.9
Statistics	16.5

**Table 2:** Distribution of majors in sample.

Major Category	% of Sample
Social or behavioral science	35.3
STEM	27.7
Business, econ, or finance	13.7
Humanities, arts, and undecided	23.3

### Attrition and missing data

**Figure 1:**
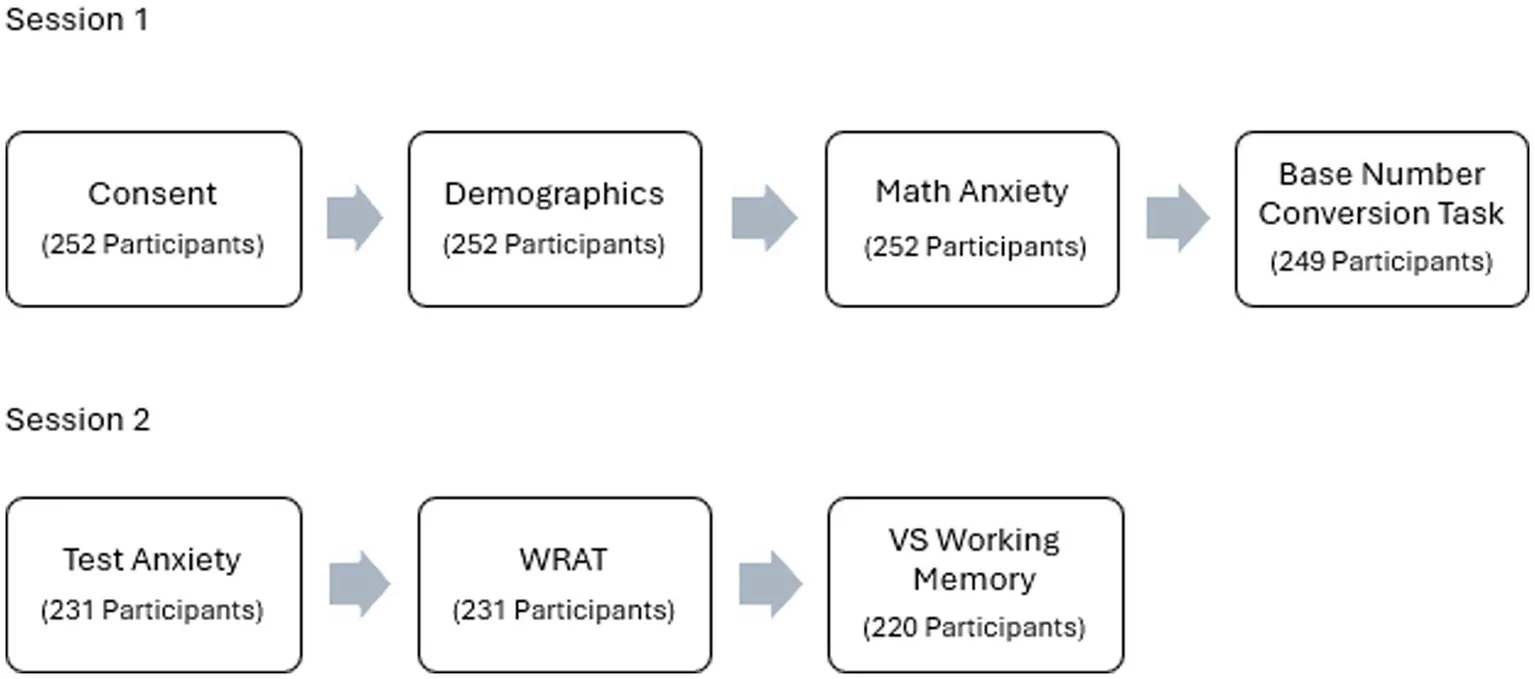
Procedure flow chart with sample sizes for each measure.

Although the full sample of 249 was included in the analysis, only 233 participants returned for session II, representing a 7.23% attrition rate. Only 220 participants had data for the visuospatial working memory capacity task due to a computer program error. Three individuals who returned for session 2 were missing WRAT and/or test anxiety scores; see Figure 1 under the procedures section for sample size for each measure. See statement on missing data in the statistical analysis section for detailed information on how missing data were handled.

### Design

The current study had a mixed experimental-correlational, between-subjects design consisting of one randomized independent variable (study strategy) and two continuous independent variables (math anxiety and math competence). A sign-in sheet with a randomized condition order was used to randomly assign participants upon arrival to one of four study strategies (described in full detail in the base number conversion task section below): full examples (*n* = 57), worked examples (*n* = 64), faded examples (*n* = 61), and practice problems (*n* = 67). The total number correct on a base number conversion test was the dependent variable. Gender, age, test anxiety, and visuospatial working memory were included as covariates. The study was conducted in the lab over two sessions, which took place between two and seven days apart.

### Materials

#### Base number conversion acquisition task

The base number conversion task consisted of a brief video lesson, a study session, and a test, which took approximately 40 min to complete. The base number conversion lesson consisted of a 10-min YouTube video on calculating base number conversions using the remainder method.^2^ In the lesson, the instructor first explained the base-10 nature of Arabic numerals, e.g., that we use 0 through 9 to represent the numbers and add additional places, such as tens or hundredths place, to represent larger numbers. The instructor then generalized the explanation to other number systems and worked through several examples of converting numbers in base 10 into an alternate base (e.g., base 2). All participants were presented with a printout with step-by-step details of the remainder procedure to refer to during the video and add notes to.

After participants watched the video, they were presented with a set of eight new base number conversion problems along with their notes from the video. Participants had a maximum of 10 min to solve or study each problem/example presented. The time remaining for each practice problem was not visible to the participant. When the time was up, the task would proceed to the next problem. Participants were provided with scratch paper to solve the problems, then entered their final answers for each problem into the Qualtrics form. The three practice problems completed by all participants, except those in the example-only condition, were scored based on correctness. They received a 1 for each problem they got correct, with a maximum total score of 3. Students’ scratch paper was not assessed, nor was partial credit given.

##### Worked example

Participants assigned to the worked example group studied five worked-out example problems and completed three practice problems independently. Worked out examples presented a step by step breakdown of the conversion procedure; see Appendix B item #1 for an example.

##### Active problem solving

Participants assigned to the active problem solving group were presented with eight practice problems to be solved independently, see Appendix B item #13 for an example.

##### Faded examples

The faded example condition is a hybrid between worked example and active problem-solving conditions, with the first five problems starting as fully worked out but with subsequent problems requiring more independent engagement as they progressed, with the last three problems to be solved independently; see Appendix 2 for all faded example stimuli.

##### Example-only

The example-only condition only consisted of eight worked out problems and required no problem solving from the participant. See the Appendix A for all practice problems presented.

#### Base number conversion test

Once all eight practice/example problems were presented, participants had their notes and practice scratch paper removed and were then given a five-minute break during which they were to complete a bird-themed word search. After the break, the computer presented instructions for the self-paced base number conversion test, participants did not have notes to reference. The test consisted of 10 new base number conversion problems, converting numbers in base 10 to either base 5, 6, 7, 8, 16, or 20. See Appendix 1 for base number conversion problems. Participants were provided with scratch paper to solve the problems, then entered their final answers for each problem into the Qualtrics form. Each problem was scored based on correctness. They received a 1 for each problem they got correct, with a maximum total score of 8. Students’ scratch paper was not assessed, nor was partial credit given.

#### Math competence

The math competence measure was a composite consisting of the Wide Range of Achievement Test (WRAT) 4 math computation subtest, students’ major, and students’ reported last math course completed. This composite was created to account for general math competence so that we do not rely on the performance on a single math assessment. The benefits of composite scores include reducing measurement error, multicollinearity, and the number of variables in the final model (Moreau and Wiebels, 2021). The WRAT math subtest consists of 40 basic mathematics questions (ranging from addition to algebra) ordered by difficulty. The WRAT is a normed, reliable, and validated measure of general math performance (see Wilkinson and Robertson, 2006). Individuals are read the instructions from the WRAT packet by the researcher and given 15 min to complete as many problems as possible. If they completed all 40 problems before the 15 min were up, they were asked to check their work.

The composite measure of math competence was created by first dummy coding students’ majors and last math courses. For students’ majors, STEM or School of Business majors were coded as 1, and all other majors were coded as 0. For students’ last math course, those who reported taking calculus 1 or higher were coded with a 1, and all else was coded with a 0. The WRAT score, major score, and last math class score were then all standardized, and an average of the three standardized measures was used as the math competence composite score.

#### Visuospatial working memory

The computerized symmetry span task from Redick et al. (2012) was used to assess visuospatial working memory. Participants are presented with a 4×4 matrix, of which various sectors are highlighted one at a time. Participants are to memorize which sectors of the matrix were highlighted as well as the order in which they were presented. As they are holding the matrix pattern in mind, they are presented with an additional static pattern for which they are to judge symmetry. The task begins with highlighting three sections of the matrix and becomes increasingly difficult until the individual makes a pre-specified number of mistakes, which indicates WM capacity is reached (see Redick et al., 2012). The task took approximately 15 min to complete, but completion time varied by individual.

#### Survey measures

##### Math anxiety

The abbreviated Mathematics Anxiety Scale (AMAS) is a shortened nine-item math anxiety questionnaire adapted by Hopko et al. (2003) from the original 98-item Mathematics Anxiety Scale by Richardson and Suinn (1972). Participants were instructed to use a scale of 1 = “not anxious at all” to 5 = “very much anxious” to rate how anxious a given situation made them feel. For example, “Taking an examination in a math course.” Cronbach’s α = 0.91.

##### Test anxiety

The four-item test anxiety subscale of the Motivated Strategies for Learning Questionnaire (Pintrich and De Groot, 1990) was used to assess participants’ test anxiety. Participants were instructed to choose a math course to complete the items about, then to rate the items based on their behavior in the class they selected on a 7-point scale, where 1 = “not at all true of me” and 7 = “very true of me.” For example, “I am so nervous during a test that I cannot remember facts I have learned.” Cronbach’s α = 0.86.

##### Other measures

Several other measures were collected as part of a larger study, but are not relevant to this current study. Participants completed the eight-item Theories of Intelligence (Dweck, 1999), the 18-item Need for Cognition (Cacioppo and Petty, 1982), and the other subscales of the MSLQ from Pintrich and De Groot (1990); specifically self-efficacy, intrinsic value, cognitive strategy use, and self-regulation. In addition, we created measures of metacognition, study strategy use, and anxiety specific to the base number conversion task.

### Procedure

The current study was conducted over two sessions spaced two to 7 days; see Figure 1. Upon arriving at session 1, participants completed informed consent and were then led into the experiment room. The experimenter would pull up the appropriate Qualtrics link on the computer for the assigned condition and enter the participant’s subject number. Participants then entered their demographics and completed the math anxiety measure. The participant would then complete the base number conversion task as described above. After the base number conversion task was completed, participants were then thanked for their participation, reminded of their follow-up appointment, and handed an appointment slip with the date and time of their session 2. Session 1 generally took 45 min to an hour to complete.

Two to 7 days after session 1, participants returned to complete the test anxiety scale, the visuospatial working memory task, and the WRAT (in the order listed). After all measures were completed, participants were thanked for their time and given a debriefing sheet that explained the study aims and provided some references. Session 2 generally took 30 to 45 min to complete.

### Statistical analyses

#### Missing data

A series of logistic regressions was run to determine if there were any significant predictors of missing data. To assess missing visuospatial working memory data we ran a model which included age, gender, test anxiety, math competence, math anxiety, final base number conversion test, and conditions (dummy coded with reference to the example only group) as predictors. The model was not significant χ^2^ (9, *N* = 230) = 16.08, *p* = 0.07 and none of the variables significantly predicted whether visuospatial working memory data was missing. Similarly to assess attrition, we ran a model with age, gender, math anxiety, final base number conversion test, and conditions (dummy coded with reference to the example only group) as predictors. The model was not significant χ^2^ (7, *N* = 249) = 10.35, *p* = 0.17 and none of the variables significantly predicted attrition. Since missing data were was not associated with any of the predictors, missing data was excluded pairwise in all analyses except for the mediation.

#### Group differences

A standard one-way ANOVA was used to test for significant differences between study strategy conditions on test anxiety, visuospatial working memory, math competence, and math anxiety. The chi-squared test of independence was used to test for differences between study strategy conditions in gender and previous experience with base number conversions. Independent samples t-tests were used to assess for differences between those who reported having learned base number conversions before and those who did not. All significance tests reported are two-tailed.

#### Performance on practice problems

A bootstrapped one-way ANOVA with 5,000 samples using Bca comparing the faded example, worked example, and active problem-solving groups (examples-only group was excluded as they did not complete any practice problems) was used to assess whether there were significant differences on the practice problems. Bootstrapping was used due to the non-normal distribution of the outcome variable.

#### Performance on test

A four-level hierarchical linear regression was used to assess the contributions of the covariates (age, gender, test anxiety, and visuospatial working memory), math competence, math anxiety, study strategies, and various interactions on the base number conversion test. Analyses were conducted with SPSS version 29 using the Enter method. The specific variables entered at each level will be discussed further in the results section. Variables included in interactions were centered if they were not standardized before interaction variables were calculated.

#### Math competence mediation

An exploratory *Post Hoc* simple mediation analysis was conducted using Andrew Hayes’ PROCESS v5 macro for SPSS using model 4 with 5,000 bootstrap samples (Hayes, 2022). The model tested whether math competence mediated the relationship between math anxiety and performance on the base number conversion test.

## Results

### Group differences and descriptive statistics

To verify success of random assignment there were no significant differences between study strategy conditions in test anxiety [*F* (3,227) = 0.39, *p* = 0.76, η_2_ = 0.01], visuospatial working memory [*F* (3,216) = 0.77, *p* = 0.51, η_2_ = 0.01], math competence [*F* (3,227) = 1.02, *p* = 0.39, η_2_ = 0.01], or math anxiety [*F* (3,245) = 0.18, *p* = 0.91, η_2_ = 0.00]. See Table 3 for overall and group descriptive statistics. There were no significant differences in the distribution of gender across the four study strategy conditions, χ^2^ (3, *N* = 249) = 2.02, *p* = 0.57.

**Table 3:** Means and standard errors by study strategy condition.

Measure	Sample size (N)	Overall M(SE)	Worked example M(SE)	Active problem Solving M(SE)	Faded example M(SE)	Example only M(SE)
Base Number Practice # Correct	249	1.91 (0.08)	2.05 (0.13)	1.73 (0.12)	1.97 (0.15)	NA
Base Number Test # Correct	249	6.32 (0.20)	6.58 (0.40)	6.28 (0.35)	6.23 (0.41)	6.16 (0.44)
Math Anxiety Sum	249	25.04 (0.47)	25.36 (0.97)	24.48 (0.89)	25.21 (0.96)	25.16 (1.00)
Math Competence	231	0.00 (0.05)	0.06 (0.09)	0.09 (0.10)	-0.04 (0.09)	−0.14 (0.11)
Test Anxiety Sum	231	15.69 (0.39)	15.57 (0.76)	16.23 (0.75)	15.10 (0.78)	15.88 (0.83)
Visuospatial Working Memory	220	20.23 (0.60)	20.63 (1.19)	20.09 (1.16)	21.22 (1.12)	18.67 (1.38)

Sixteen participants reported they had learned base number conversions previously, despite the prescreening. There were no significant differences between those who reported that they learned base number conversions previously and those who did not on the base number conversion test [*t* (247) = −0.42, *p = 0*.68, *d* = −0.11], math anxiety [*t*(247) = −1.58, *p = 0*.12, *d* = −0.42], math competence [*t* (229) = 0.56, *p = 0*.58, *d* = 0.16], visuospatial working memory [*t* (218) = 1.42, *p = 0*.16, *d* = 0.40], or test anxiety [*t* (229) = 0.30, *p = 0*.77, *d* = 0.08]. There was also no significant difference in the number of those who reported they had learned base number conversions previously between the four study strategy conditions, χ^2^ (3, *N* = 249) = 1.73, *p* = 0.63.

### Performance on practice problems

There were no significant differences between the three study strategy conditions on the practice problems [*F* (2,191) = 1.55, *p* = 0.22, η_2_ = 0.02]; see row 1 in Table 1 for group means and standard errors. The BCa 95% confidence intervals for the group means were [1.77,2.31], [1.68,2.24], and [1.49,1.97] for the worked example, faded example, and active problem solving groups, respectively.

### Performance on test

A four-level hierarchical linear regression was used to assess the unique contribution of math anxiety and study strategies to predicting base number conversion test performance. Visual inspection of scatterplots indicated linear relationships between predictors and the outcome. Examination of standardized residuals suggested homoscedasticity and approximate normality. The Durbin–Watson statistic (*DW* = 1.98) indicated independence of residuals. Multicollinearity was not evident, with tolerance values greater than 0.20 and variance inflation factors below 5. Model 1 included all covariates specified above in addition to math competence as predictors of base number test performance. Model 1 was significant *F* (5,212) = 9.40, *p* < 0.001, *R*^2^ = 0.18. Only math competence was a significant predictor of performance on the base number conversion test, *β* = 0.38, *t* (229) = 5.70, *p <* 0.001, *sr*^2^ = 0.13, with higher math competence associated with higher performance on the base number conversion test. Model 2 included all the variables from Model 1, plus math anxiety and the math anxiety X math competence interaction. Model 2 added significant variance *F_change_* (2,210) = 6.03, *p* = 0.003, *R*^2^_change_ = 0.04. math competence was still a significant predictor β = 0.32, *t* (229) = 4.64, *p <* 0.001, *sr*^2^ = 0.08 in Model 2. In addition, Math Anxiety β = −0.19, *t* (247) = −2.64, *p =* 0.009, *sr*^2^ = 0.03 was also a significant predictor. Math anxiety was negatively associated with performance on the base number conversion test, with higher math anxiety associated with lower performance on the base number conversion test. Model 3 included all previous predictors in addition to the study strategy conditions, dummy coded with the examples only condition as the reference group. Model 3 did not explain any additional variance compared to Model 2, *F*_change_ (3,207) = 0.22, *p* = 0.88, *R*^2^_change_ = 0.003. Model 4 included all previous predictors and added the interactions between math anxiety and each of the study strategy conditions. Model 4 did not explain any additional variance compared to Model 3, *F*_change_ (3,204) = 0.86, *p* = 0.46, *R*^2^_change_ = 0.01. See Table 4 for full model values.

**Table 4:** Hierarchical linear regression values predicting base number conversion test performance.

	Block 1							Block 2						
Predictor	B	*SE* B	Wald	*p*	β	95% CI B		B	*SE* B	Wald	*p*	*β*	95% CI B	
Age	−0.01	0.05	−0.22	0.83	−0.02	−0.10	0.08	−0.02	0.05	−0.35	0.73	−0.02	−0.10	0.07
Female	−0.12	0.40	−0.30	0.77	−0.02	−0.92	0.67	0.01	0.40	−0.02	0.99	0.00	−0.79	0.77
Test anxiety	0.01	0.03	0.35	0.73	0.02	−0.06	0.08	0.05	0.04	1.32	0.19	0.09	−0.02	0.12
Spatial WM	0.04	0.02	1.96	0.05	0.13	0.00	0.09	0.04	0.02	1.79	0.08	0.11	0.00	0.08
Math competence	**1.57**	**0.27**	**5.70**	**<0.001**	**0.38**	**1.02**	**2.11**	**1.30**	**0.28**	**4.64**	**<0.001**	**0.32**	**0.75**	**1.85**
Math anxiety	–	–	–	–	–	–	–	**−0.08**	**0.03**	**−2.64**	**0.01**	**−0.19**	**−0.14**	**−0.02**
Math anxiety x math competence	–	–	–	–	–	–	–	0.06	0.03	1.91	0.06	0.12	0.00	0.12
	*F*(5,212) = 9.40, *p* < 0.001, *R*^2^ = 0.18							*F*_change_(2,210) = 6.03, *p* = 0.003, *R*^2^_change_ = 0.04						

	Block 3							Block 4						
Predictor	B	*SE* B	Wald	*p*	β	95% CI B		B	*SE* B	Wald	*p*	β	95% CI B	
Age	−0.02	0.05	−0.40	0.69	−0.03	−0.11	0.07	−0.02	0.05	−0.33	0.74	−0.02	−0.11	0.08
Female	0.02	0.40	0.05	0.96	0.00	−0.77	0.81	0.03	0.40	0.08	0.94	0.01	−0.76	0.82
Test Anxiety	0.05	0.04	1.33	0.19	0.09	−0.02	0.12	0.05	0.04	1.38	0.17	0.10	−0.02	0.12
Spatial WM	0.04	0.02	1.78	0.08	0.11	−0.00	0.08	0.04	0.02	1.80	0.07	0.12	0.00	0.09
Math Competence	**1.30**	**0.28**	**4.59**	**<0.001**	**0.32**	**0.74**	**1.90**	**1.27**	**0.28**	**4.48**	**<0.001**	**0.31**	**0.71**	**1.83**
Math Anxiety	**−0.08**	**0.03**	**−2.67**	**0.01**	**−0.19**	**−0.14**	**−0.02**	−0.10	0.06	−1.87	0.06	−0.25	−0.21	0.01
Math Anxiety x Math Competence	0.06	0.03	1.89	0.06	0.12	0.00	0.12	0.07	0.03	2.08	0.04	0.13	0.00	0.13
Active Problem Solving	−0.32	0.55	−0.58	0.56	−0.05	−1.40	0.76	−0.35	0.55	−0.63	0.53	−0.05	−1.43	0.73
Faded Examples	−0.19	0.56	−0.34	0.73	−0.03	−1.30	0.92	−0.20	0.56	−0.36	0.72	−0.03	−1.31	0.91
Worked Examples	0.08	0.55	0.14	0.89	0.01	−1.01	1.16	0.06	0.55	0.11	0.91	0.01	−1.03	1.15
Math Anxiety × Active Problem Solving	–	–	–	–	–	–	–	−0.03	0.07	−0.47	0.64	−0.04	−0.18	0.11
Math AnxietyXFaded Examples	–	–	–	–	–	–	–	−0.04	0.07	0.89	0.38	0.08	−0.08	0.21
Math Anxiety × Worked Examples	–	–	–	–	–	–	–	0.06	0.08	0.77	0.44	0.07	−0.09	0.21
	*F*_change_(3,207) = 0.22, *p* = 0.88, *R*^2^_change_ = 0.003							*F*_change_(3,203) = 0.86, *p* = 0.46, *R*^2^_change_ = 0.01						

### Math competence mediation

**Figure 2:**
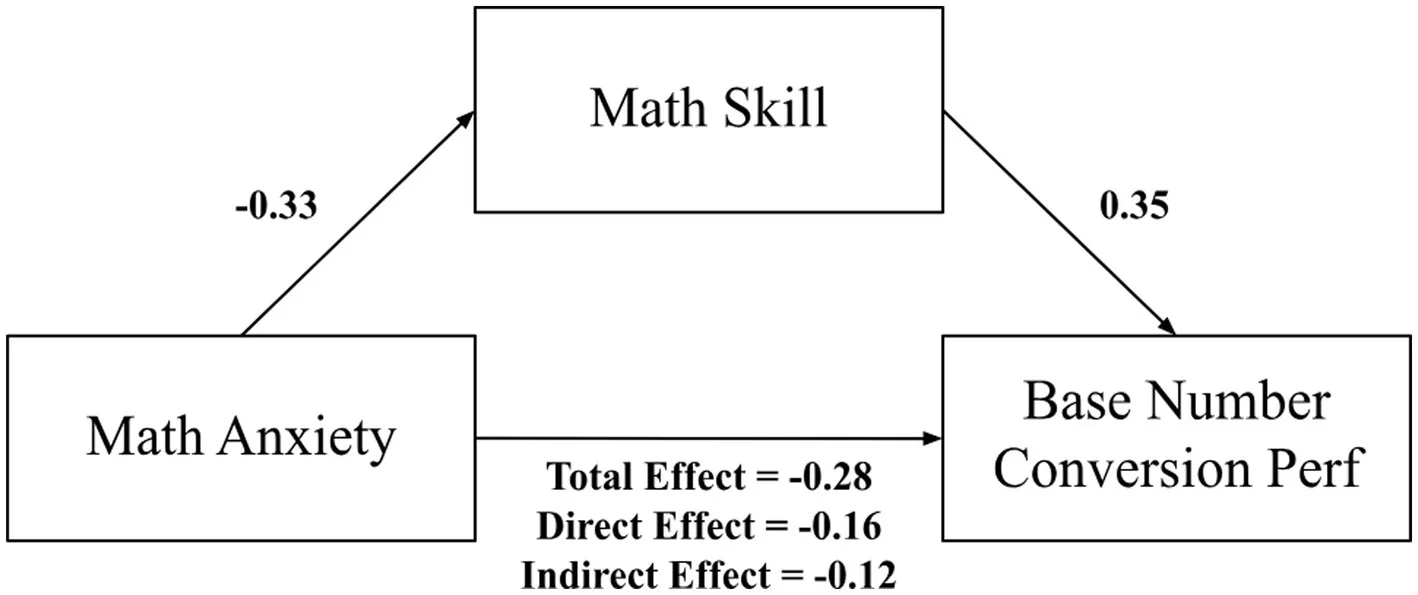
Math competence mediation of math anxiety and base number conversion test.

We were curious whether math competence mediated the relationship between math anxiety and performance on the base number conversion test. Beta’s reported are standardized values. The total effect of math anxiety on the base number conversion test was significant, b = −0.28, SE = 0.06, *t* (229) = −4.36, *p* < 0.001, 95% CI [−0.40, −0.15]. When math competence was included in the model, the direct effect of math anxiety on base number conversion performance remained significant, b = −0.16., SE = 0.06, *t* (228) = −2.52, *p* = 0.012, 95% CI [−0.28,−0.04]. The indirect effect of math anxiety on base number conversion performance through math competence was significant, b = −0.12, SE_bootstrapped_ = 0.03, 95% BCa [−0.19, −0.07], indicating partial mediation. The current analysis does not assess directional or causal relationships. See Figure 2 for the mediation diagram.

## Discussion

The objectives of the study were twofold: to assess the extent to which math anxiety relates to new math skill acquisition, above and beyond individuals’ existing basic math competence, and to explore whether the strategy used while studying the novel information interacts with the individuals’ math anxiety to achieve different outcomes. Here we found that math anxiety was negatively associated with the performance of a novel math task above and beyond individuals’ math competence. We also found that individuals’ math competence partially mediated the relationship between math anxiety and performance on the novel math task, with math anxiety having both direct and indirect relationships with performance on the novel math task. The current study was inconclusive on the differences between study strategies or with their interaction with math anxiety.

### Math anxiety and novel math skills

These findings suggest that math anxiety is related to impaired performance on newly acquired mathematical information that cannot be accounted for by math competence. As discussed previously, evidence suggests math anxiety leads individuals to underperform on measures of preexisting math skill (Ashcraft and Moore, 2009). It is possible that math anxiety is associated with a greater disruption in performance of newly acquired math tasks than with preexisting and well learned tasks; however, it is also possible that math anxiety is interfering with the acquisition of the task itself, or both things could be true. The current study cannot tease these two apart, but it does provide evidence that math anxiety has a unique relationship with performance of a new math skill, and future studies should further explore the nature of this relationship. Based on the Disruption Account (Ramirez et al., 2018), it could be that as learning new information is more cognitively demanding than retrieving existing knowledge, individuals with high math anxiety are more prone to the negative disruptive cognitive effects of math anxiety when learning new math material. According to the processing efficiency theory (Eysenck and Calvo, 1992), if individuals with high math anxiety are preoccupied with worries about their performance, they will have fewer working memory resources available to dedicate to the learning of the new material. This may slow down their ability to encode and create an accurate mental representation and achieve fluency with the new procedure, making them more prone to errors. In contrast, when dealing with pre-existing knowledge, presumably the mental representation already exists; therefore, math anxiety only interferes with the retrieval of the information. It is also possible that some unassessed low-level numerical deficit, not captured by our math competency composite, could directly or indirectly account for the relationship between math anxiety and the acquisition of novel math skills.

This finding adds complexity to our current understanding of how math anxiety is associated with individuals opting out of higher math education. It provides evidence that it is not merely that individuals with high math anxiety tend to score lower on measures of achievement, but that they are also likely to perform even worse on newly acquired information compared to their less anxious counterparts with the same level of competency. Most math curricula build from previous math skills and knowledge. If individuals with high math anxiety do not master certain math topics, they are likely up for a difficult path as topics become more and more complex. These findings suggest that we need to focus our interventions not only on reducing the negative performance effects of math anxiety but potentially also trying to mitigate math anxiety during the acquisition process itself.

### Study strategies and math anxiety

Previous research on the use of worked examples showed that the two strategies could offer different benefits; however, our current study was inconclusive. Overall, whether a student studied examples, completed faded examples, studied some examples and completed some practice problems, or only completed practice problems after initial instruction, they all seemed equally effective regardless of individual differences in visuospatial working memory, math competency, or math anxiety. Originally, we surmised that worked examples could help reduce cognitive load during study (Cooper and Sweller, 1987; Sweller et al., 1990; Sweller and Cooper, 1985; Tuovinen and Sweller, 1999), but that active problem solving could provide desirable difficulties (Bjork, 1994; Bjork and Bjork, 2020; McNamara and Healy, 1995, 2000; Rittle-Johnson and Kmicikewycz, 2008). Whether one strategy was more beneficial than the other would depend on whether an individual’s cognitive resources were overloaded. One potential reason we did not find any differences between strategies or interactions with math anxiety is that we had a fairly high math achieving sample. Our sample consisted of undergraduate students admitted to a very selective university; 66.7% of the sample reported that they took calculus 1 or higher as their last math course. It is possible that for a sample that is very high math achieving, the different study strategies do not matter, as was found by Rittle-Johnson and Kmicikewycz (2008). It is also possible that there was a lack of distinctiveness between conditions, that all groups were presented with enough worked out examples between the video and the study sheet that made the differences between the conditions irrelevant. This could be especially true for this high achieving sample. Future studies that closely assess the acquisition process and students’ progress throughout the topic would better shed light on whether the different study strategies have bearing, especially when considering their interaction with math anxiety.

### Limitations and future directions

Our current study had several strengths. First, it used a novel math task and multi-stage procedure that mimics the typical math skill acquisition process. Second, it controlled for individuals’ math competency using a composite score that consisted of the students’ past math progress and their basic math skills. Third, it controlled for visuospatial working memory capacity and test anxiety, both known factors in the math anxiety-math performance relationship. Together, these features increase our confidence in the validity of our findings.

Despite these strengths, there are several limitations to the current study. First, the current study design does not allow for any causal conclusions; longitudinal data would be needed. Second, we cannot determine whether math anxiety is negatively associated with acquisition of the math task or just performance on the novel math task; study designs that could directly assess online working memory load and directly measure progress during acquisition of the new content would be needed. Third, the sample is not diverse enough in math ability and the task potentially not distinctive enough between conditions to allow for any conclusions about whether or not study strategies could interact with math anxiety to influence the acquisition of new math skills. Fourth, we did not account for other variables that are known to relate to math anxiety and math performance, e.g., self-efficacy (Jain and Dowson, 2009). Lastly, it is important to recognize that the measurement for the base number conversion task was limited, as it was a sum score reflecting only the number of problems correct; more robust measures would allow for more nuanced analyses in the future. Notwithstanding these limitations, this study demonstrated an important initial finding which opens the door for future studies to further expand our understanding of the role of math anxiety in the acquisition of new math skills and knowledge.

## Conclusion

In conclusion, we found evidence that math anxiety uniquely relates to poorer performance of new math material, above and beyond general math competency. This could be explained by increased interference by math anxiety on performance of new content, or it could be due to interference with the learning process itself, or through some other alternate explanation. In addition, data suggest that math anxiety has both a direct and indirect relationship with performance on a newly acquired math task, which is mediated by math competence. Future research directly assessing the acquisition process is needed to elucidate the mechanisms by which math anxiety relates to the acquisition of new math skills.

## Data Availability

The raw data supporting the conclusions of this article will be made available by the authors, without undue reservation.

## Footnotes

2 see the following link for the original video here https://www.youtube.com/watch?v=TjvexIVV_gI
