# Supplementary material for: Math anxiety predicts initial acquisition of new math skills over and above math competence and study strategy
Source: Front Psychol. 2026 Sep 8;17:1829468. doi: 10.3389/fpsyg.2026.1829468 (PMC13593877; doi:10.3389/fpsyg.2026.1829468)
Supplement: Supplementary file 1 [file Supplementary_file_1.docx]

**Appendix A Base Number Conversion Problems**

| **Practice Problems** |  | **Test Problems** |  |  |  |  |  |  |  |  |  |
| --- | --- | --- | --- | --- | --- | --- | --- | --- | --- | --- | --- |
| 510 base 2 |  | 14_10_ base 7 |  |  |  |  |  |  |  |  |  |
| 810 base 3 |  | 16_10_ base 6 |  |  |  |  |  |  |  |  |  |
| 1010 base 4 |  | 31_10_ base 7 |  |  |  |  |  |  |  |  |  |
| 610 base 2 |  | 33_10_ base 6 |  |  |  |  |  |  |  |  |  |
| 65510 base 20 |  | 35_10_ base 5 |  |  |  |  |  |  |  |  |  |
| 910 base 3 |  | 39_10_base 8 |  |  |  |  |  |  |  |  |  |
| 7_10_ base 4 |  | 43_10_base 8 |  |  |  |  |  |  |  |  |  |
| 29_10_ base 16 |  | 45_10_ base 16 |  |  |  |  |  |  |  |  |  |
|  |  | 47_10_ base 5 |  |  |  |  |  |  |  |  |  |
|  |  | 721_10_ base 20 |  |  |  |  |  |  |  |  |  |

**Appendix B Faded Example Condition Stimuli**

1) 2)

3) 4) 5) 6) 7) 8) 9) 10)

11) 12)

13) 14)

15)
